# 7a-Phenyl­tetra­hydro­pyrrolo­[2,1-*b*]oxazol-5(6*H*)-one

**DOI:** 10.1107/S2414314620009190

**Published:** 2020-07-10

**Authors:** Elena I. Linkova, Vyacheslav S. Grinev, Oksana A. Mayorova, Alevtina Yu. Yegorova

**Affiliations:** a Institute of Chemistry, N.G. Chernyshevsky National Research Saratov State University, Ulitsa Astrakhanskaya, 83, Saratov 410012, Russian Federation; b Institute of Biochemistry and Physiology of Plants and Microorganisms, Russian Academy of Sciences, 13 Prospekt Entuziastov, Saratov 410049, Russian Federation; University of Aberdeen, Scotland

**Keywords:** crystal structure, pyrrolo­[2,1-*b*]oxazol-5(6*H*)-one, C—H⋯O contacts, C—H⋯π contacts

## Abstract

In the title compound, C_12_H_13_NO_2_, the pyrrolidinone moiety is almost flat while the oxazole ring adopts an envelope conformation with the carbon atom bearing the phenyl substituent as the flap: the angle between the mean planes of the fused heterocyclic rings is 45.47 (19)°. In the crystal, C—H⋯O and C—H⋯π contacts link the mol­ecules into infinite [010] chains.

## Abstract 2

In the crystal of the title compound, C—H⋯O and C—H⋯π contacts link the mol­ecules into infinite chains directed along the *b*-axis direction.

## Graphical abstract

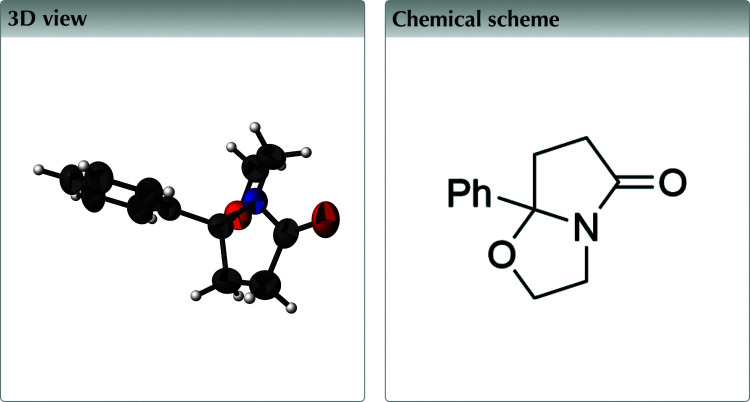

## Structure description

The title compound, C_12_H_13_NO_2_, has been reported in the literature several times (Aeberli & Houlihan, 1969 ▸; Aeberli *et al.*, 1976 ▸; Amal’chieva & Egorova, 2006 ▸). It has been also reported for its anti-depressant (Aeberli *et al.*, 1976 ▸) and anti-convulsant activities (Trapani *et al.*, 1996 ▸) as well as the synthetic potential to obtain 4,5-di­hydro-2*H*-pyridazin-3-ones (Lim *et al.*, 2003 ▸). We now describe its crystal structure.

**Figure 1:**
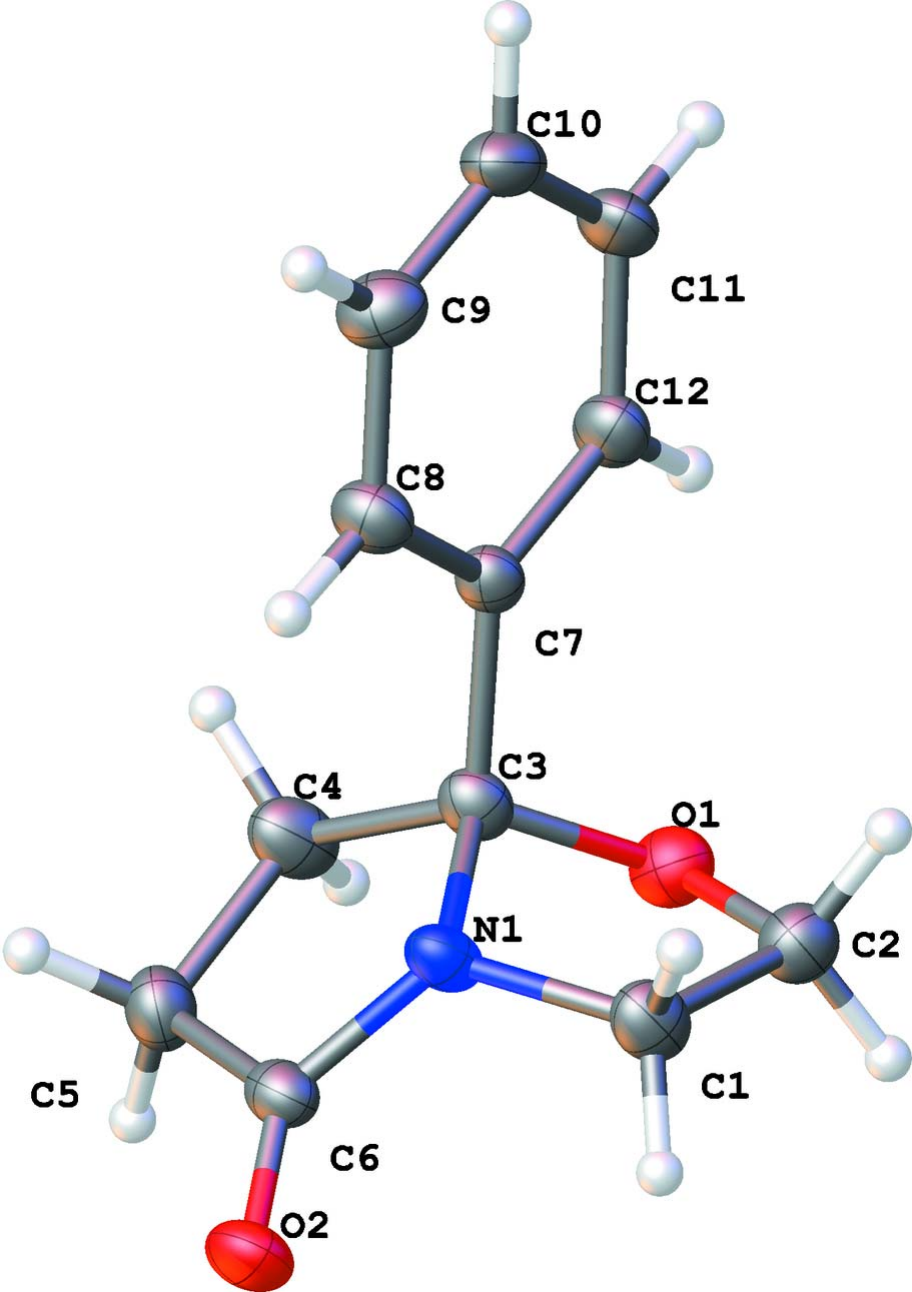
The mol­ecular structure of the title compound showing 50% displacement ellipsoids.

Mol­ecules of title compound consist of pyrrolidinone and oxazole rings fused *via* the C3—N1 edge into a bicyclic system (Fig. 1 ▸). The pyrrolidinone moiety is almost flat (r.m.s. deviation = 0.054 Å) with a maximum torsion angle N1—C6—C5—C4 of 13.4 (5)°, whereas the minimum torsion angle C5—C4—C3—N1 is 2.7 (5)°. The oxazole ring is more twisted and adopts an envelope conformation with atom C3 as the flap and a maximum torsion angle C2—O1—C3—N1 of −35.7 (4)°. The heterocyclic rings are fused with a dihedral angle between their mean planes of 45.47 (19)°. The phenyl substituent is located orthogonally to the mean plane of the whole bicycle [dihedral angle = 89.28 (14)°].

**Figure 2:**
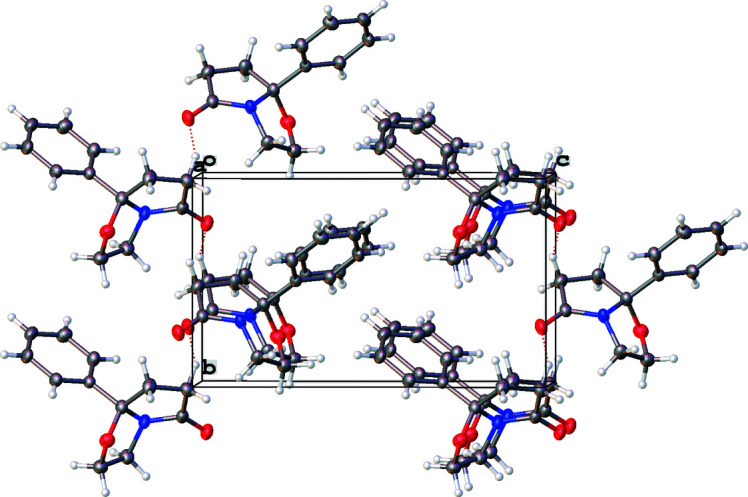
The packing of the title compound viewed down [100] showing hydrogen bonds as red dashed lines.

**Figure 3:**
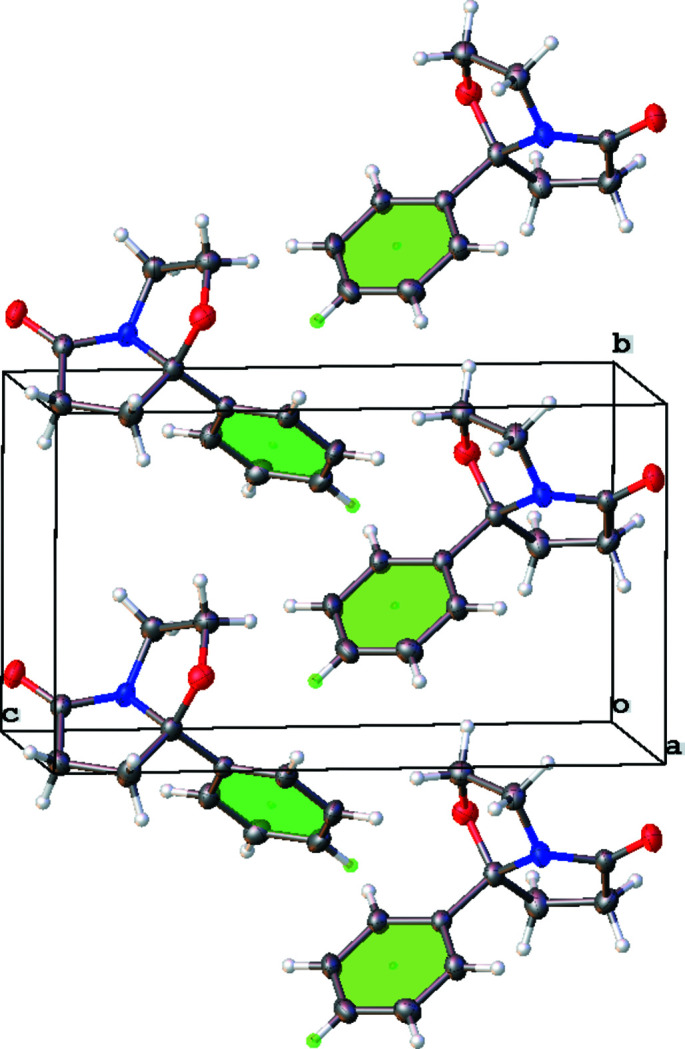
The packing of the title compound showing C—H⋯π inter­actions.

In contrast to closely related pyrrolo­pyrimidino­nes (Grinev *et al.*, 2020 ▸), there is no classical hydrogen bonding in the crystal of the title mol­ecule, obviously due to the absence of NH groups (Fig. 2 ▸). The mol­ecules are connected *via* weak C5—H5*A*⋯O2 links (Table 1 ▸) to generate infinite chains directed along [010]. The H5*A*⋯O2 distance of 2.58 Å is significantly longer than the corresponding distance in pyrrolo­pyrimidino­nes [2.28 (5)–2.306 (18) Å]. Moreover, there are C10—H10⋯π contacts to an adjacent phenyl ring (Fig. 3 ▸), which reinforce the [010] chains.

## Synthesis and crystallization

5-Phenyl­furan-2(3*H*)-one (1 g, 6 mmol) and ethano­lamine (0.34 g, 6 mmol) were placed in a round-bottomed flask equipped with Dean–Stark apparatus. Dry benzene (30 ml) was added and the reaction mixture refluxed for 3–4 h. After being left to stand overnight, the separated crystals and precipitate were washed with benzene and acetone and the solid placed in a vacuum desiccator for drying (yield 0.91 g, 75%; m.p. 65–67°C). The single crystal used for data collection was obtained directly from the cooled reaction mixture.

## Refinement

Crystal data, data collection and structure refinement details are summarized in Table 2 ▸.

## Supplementary Material

Crystal structure: contains datablock(s) I. DOI: 10.1107/S2414314620009190/hb4353sup1.cif

Structure factors: contains datablock(s) I. DOI: 10.1107/S2414314620009190/hb4353Isup2.hkl

Supporting information file. DOI: 10.1107/S2414314620009190/hb4353Isup3.cml

CCDC reference: 2014300

Additional supporting information:  crystallographic information; 3D view; checkCIF report

## Figures and Tables

**Table 1:** Hydrogen-bond geometry (Å, °) *Cg*3 is the centroid of the C7–C12 ring.

*D*—H⋯*A*	*D*—H	H⋯*A*	*D*⋯*A*	*D*—H⋯*A*
C5—H5*A*⋯O2^i^	0.97	2.58	3.346 (6)	136
C10—H10⋯*Cg*3^ii^	0.93	2.88	3.734 (6)	154

Symmetry codes: (i) 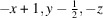 ; (ii) 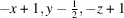 .

**Table 2:** Experimental details

Crystal data	
Chemical formula	C_12_H_13_NO_2_
*M*_r_	203.23
Crystal system, space group	Monoclinic, *P*2_1_
Temperature (K)	295
*a*, *b*, *c* (Å)	5.7173 (17), 7.346 (3), 12.436 (4)
β (°)	93.07 (3)
*V* (Å^3^)	521.5 (3)
*Z*	2
Radiation type	Mo *K*α
μ (mm^−1^)	0.09
Crystal size (mm)	0.55 × 0.1 × 0.08

Data collection	
Diffractometer	Agilent Technologies New Xcalibur, Ruby
Absorption correction	Multi-scan (*CrysAlis PRO*; Agilent, 2014 ▸)
*T*_min_, *T*_max_	0.217, 1.000
No. of measured, independent and observed [*I* > 2σ(*I*)] reflections	5059, 2406, 1462
*R*_int_	0.048
(sin θ/λ)_max_ (Å^−1^)	0.691

Refinement	
*R*[*F* ^2^ > 2σ(*F* ^2^)], *wR*(*F* ^2^), *S*	0.054, 0.160, 1.06
No. of reflections	2406
No. of parameters	137
No. of restraints	1
H-atom treatment	H-atom parameters constrained
Δρ_max_, Δρ_min_ (e Å^−3^)	0.16, −0.15

Computer programs: *CrysAlis PRO* (Agilent, 2014 ▸), *SHELXS* (Sheldrick, 2008 ▸), *SHELXL* (Sheldrick, 2015 ▸) and *OLEX2* (Dolomanov *et al.*, 2009 ▸).

## full crystallographic data

### Crystal data

**Table:**

C_12_H_13_NO_2_	*F*(000) = 216
*M**_r_* = 203.23	*D*_x_ = 1.294 Mg m^−^^3^
Monoclinic, *P*2_1_	Mo *K*α radiation, λ = 0.71073 Å
*a* = 5.7173 (17) Å	Cell parameters from 1357 reflections
*b* = 7.346 (3) Å	θ = 3.5–22.6°
*c* = 12.436 (4) Å	µ = 0.09 mm^−^^1^
β = 93.07 (3)°	*T* = 295 K
*V* = 521.5 (3) Å^3^	Needle, clear colourless
*Z* = 2	0.55 × 0.1 × 0.08 mm

### Data collection

**Table:**

Agilent Technologies New Xcalibur, Ruby diffractometer	2406 independent reflections
Radiation source: Enhance (Mo) X-ray Source	1462 reflections with *I* > 2σ(*I*)
Graphite monochromator	*R*_int_ = 0.048
Detector resolution: 10.4752 pixels mm^-1^	θ_max_ = 29.4°, θ_min_ = 3.2°
ω scans	*h* = −7→7
Absorption correction: multi-scan (CrysAlisPro; Agilent, 2014)	*k* = −9→9
*T*_min_ = 0.217, *T*_max_ = 1.000	*l* = −16→11
5059 measured reflections	

### Refinement

**Table:**

Refinement on *F*^2^	Hydrogen site location: inferred from neighbouring sites
Least-squares matrix: full	H-atom parameters constrained
*R*[*F*^2^ > 2σ(*F*^2^)] = 0.054	*w* = 1/[σ^2^(*F*_o_^2^) + (0.0638*P*)^2^] where *P* = (*F*_o_^2^ + 2*F*_c_^2^)/3
*wR*(*F*^2^) = 0.160	(Δ/σ)_max_ < 0.001
*S* = 1.06	Δρ_max_ = 0.16 e Å^−^^3^
2406 reflections	Δρ_min_ = −0.15 e Å^−^^3^
137 parameters	Extinction correction: SHELXL2018/1 (Sheldrick 2015), Fc^*^=kFc[1+0.001xFc^2^λ^3^/sin(2θ)]^-1/4^
1 restraint	Extinction coefficient: 0.094 (18)
Primary atom site location: structure-invariant direct methods	

### Special details

**Table:**

Geometry. All esds (except the esd in the dihedral angle between two l.s. planes) are estimated using the full covariance matrix. The cell esds are taken into account individually in the estimation of esds in distances, angles and torsion angles; correlations between esds in cell parameters are only used when they are defined by crystal symmetry. An approximate (isotropic) treatment of cell esds is used for estimating esds involving l.s. planes.

### Fractional atomic coordinates and isotropic or equivalent isotropic displacement parameters (Å^2^)

**Table:**

	*x*	*y*	*z*	*U*_iso_*/*U*_eq_	
C1	0.4191 (7)	0.8471 (7)	0.1967 (3)	0.0669 (12)	
H1A	0.440537	0.941850	0.143766	0.080*	
H1B	0.563590	0.831027	0.240152	0.080*	
N1	0.3418 (5)	0.6763 (5)	0.1455 (2)	0.0560 (9)	
O1	0.0340 (4)	0.7581 (5)	0.2424 (2)	0.0666 (9)	
C2	0.2140 (7)	0.8897 (6)	0.2663 (3)	0.0694 (13)	
H2A	0.264333	0.883883	0.341924	0.083*	
H2B	0.155301	1.011334	0.250689	0.083*	
O2	0.4289 (5)	0.7275 (5)	−0.0288 (2)	0.0743 (10)	
C3	0.1537 (6)	0.6008 (6)	0.2049 (3)	0.0545 (10)	
C4	−0.0033 (7)	0.4983 (8)	0.1217 (3)	0.0750 (13)	
H4A	−0.017904	0.371365	0.141669	0.090*	
H4B	−0.158290	0.552282	0.115247	0.090*	
C5	0.1181 (7)	0.5165 (7)	0.0181 (3)	0.0686 (12)	
H5A	0.180468	0.399755	−0.003026	0.082*	
H5B	0.009114	0.559003	−0.038927	0.082*	
C6	0.3109 (7)	0.6501 (6)	0.0374 (3)	0.0559 (10)	
C7	0.2432 (6)	0.4828 (5)	0.2990 (3)	0.0489 (9)	
C8	0.4440 (6)	0.3789 (6)	0.2926 (3)	0.0622 (11)	
H8	0.529967	0.384189	0.231306	0.075*	
C9	0.5168 (7)	0.2675 (6)	0.3775 (4)	0.0712 (12)	
H9	0.653085	0.199387	0.373066	0.085*	
C10	0.3912 (8)	0.2559 (7)	0.4680 (4)	0.0724 (12)	
H10	0.441108	0.179917	0.524518	0.087*	
C11	0.1929 (9)	0.3565 (8)	0.4745 (3)	0.0746 (13)	
H11	0.107507	0.349304	0.535934	0.090*	
C12	0.1163 (7)	0.4699 (6)	0.3904 (3)	0.0633 (11)	
H12	−0.020343	0.537312	0.395607	0.076*	

### Atomic displacement parameters (Å^2^)

**Table:**

	*U*^11^	*U*^22^	*U*^33^	*U*^12^	*U*^13^	*U*^23^
C1	0.068 (2)	0.072 (3)	0.061 (3)	−0.019 (3)	0.005 (2)	−0.004 (2)
N1	0.0587 (17)	0.066 (2)	0.0439 (19)	−0.0115 (16)	0.0075 (13)	0.0058 (16)
O1	0.0574 (14)	0.0675 (18)	0.076 (2)	0.0055 (15)	0.0118 (13)	0.0046 (16)
C2	0.075 (3)	0.063 (3)	0.070 (3)	−0.005 (2)	0.005 (2)	0.000 (2)
O2	0.090 (2)	0.082 (2)	0.0525 (18)	−0.0011 (18)	0.0205 (15)	0.0124 (16)
C3	0.0511 (19)	0.063 (2)	0.050 (2)	−0.0077 (18)	0.0097 (16)	0.001 (2)
C4	0.075 (3)	0.088 (4)	0.061 (3)	−0.026 (3)	−0.002 (2)	0.003 (3)
C5	0.078 (2)	0.068 (3)	0.060 (2)	−0.003 (2)	0.006 (2)	−0.012 (2)
C6	0.061 (2)	0.060 (3)	0.047 (2)	0.008 (2)	0.0061 (16)	0.005 (2)
C7	0.0494 (19)	0.051 (2)	0.047 (2)	−0.0065 (17)	0.0098 (14)	0.0002 (19)
C8	0.060 (2)	0.073 (3)	0.055 (2)	−0.001 (2)	0.0119 (18)	0.006 (2)
C9	0.064 (2)	0.068 (3)	0.082 (3)	0.005 (2)	0.007 (2)	0.012 (3)
C10	0.098 (3)	0.062 (3)	0.057 (3)	−0.011 (3)	−0.002 (2)	0.013 (2)
C11	0.106 (3)	0.069 (3)	0.052 (2)	−0.003 (3)	0.028 (2)	0.009 (2)
C12	0.070 (2)	0.062 (3)	0.059 (2)	−0.003 (2)	0.0219 (18)	0.002 (2)

### Geometric parameters (Å, º)

**Table:**

C1—H1A	0.9700	C4—C5	1.502 (6)
C1—H1B	0.9700	C5—H5A	0.9700
C1—N1	1.464 (6)	C5—H5B	0.9700
C1—C2	1.527 (6)	C5—C6	1.486 (6)
N1—C3	1.447 (5)	C7—C8	1.384 (5)
N1—C6	1.361 (5)	C7—C12	1.385 (5)
O1—C2	1.432 (5)	C8—H8	0.9300
O1—C3	1.434 (5)	C8—C9	1.382 (6)
C2—H2A	0.9700	C9—H9	0.9300
C2—H2B	0.9700	C9—C10	1.370 (6)
O2—C6	1.230 (5)	C10—H10	0.9300
C3—C4	1.531 (5)	C10—C11	1.359 (7)
C3—C7	1.523 (5)	C11—H11	0.9300
C4—H4A	0.9700	C11—C12	1.389 (6)
C4—H4B	0.9700	C12—H12	0.9300
			
H1A—C1—H1B	109.3	C4—C5—H5A	110.3
N1—C1—H1A	111.5	C4—C5—H5B	110.3
N1—C1—H1B	111.5	H5A—C5—H5B	108.6
N1—C1—C2	101.5 (3)	C6—C5—C4	107.0 (4)
C2—C1—H1A	111.5	C6—C5—H5A	110.3
C2—C1—H1B	111.5	C6—C5—H5B	110.3
C3—N1—C1	108.8 (3)	N1—C6—C5	108.0 (4)
C6—N1—C1	124.8 (4)	O2—C6—N1	123.3 (4)
C6—N1—C3	112.9 (3)	O2—C6—C5	128.7 (4)
C2—O1—C3	105.2 (3)	C8—C7—C3	121.1 (3)
C1—C2—H2A	110.1	C8—C7—C12	118.8 (4)
C1—C2—H2B	110.1	C12—C7—C3	120.0 (3)
O1—C2—C1	108.0 (3)	C7—C8—H8	120.0
O1—C2—H2A	110.1	C9—C8—C7	120.0 (4)
O1—C2—H2B	110.1	C9—C8—H8	120.0
H2A—C2—H2B	108.4	C8—C9—H9	119.5
N1—C3—C4	105.6 (3)	C10—C9—C8	121.0 (4)
N1—C3—C7	112.5 (3)	C10—C9—H9	119.5
O1—C3—N1	103.8 (3)	C9—C10—H10	120.3
O1—C3—C4	110.1 (3)	C11—C10—C9	119.4 (4)
O1—C3—C7	110.8 (3)	C11—C10—H10	120.3
C7—C3—C4	113.6 (4)	C10—C11—H11	119.6
C3—C4—H4A	110.8	C10—C11—C12	120.8 (4)
C3—C4—H4B	110.8	C12—C11—H11	119.6
H4A—C4—H4B	108.9	C7—C12—C11	120.1 (4)
C5—C4—C3	104.8 (3)	C7—C12—H12	120.0
C5—C4—H4A	110.8	C11—C12—H12	120.0
C5—C4—H4B	110.8		
			
C1—N1—C3—O1	33.1 (3)	C3—O1—C2—C1	25.9 (4)
C1—N1—C3—C4	148.9 (4)	C3—C4—C5—C6	−9.5 (5)
C1—N1—C3—C7	−86.7 (4)	C3—C7—C8—C9	−177.5 (4)
C1—N1—C6—O2	32.0 (6)	C3—C7—C12—C11	177.4 (4)
C1—N1—C6—C5	−148.2 (4)	C4—C3—C7—C8	85.8 (4)
N1—C1—C2—O1	−5.7 (4)	C4—C3—C7—C12	−90.5 (4)
N1—C3—C4—C5	2.7 (5)	C4—C5—C6—N1	13.4 (5)
N1—C3—C7—C8	−34.1 (5)	C4—C5—C6—O2	−166.8 (4)
N1—C3—C7—C12	149.6 (4)	C6—N1—C3—O1	−109.9 (4)
O1—C3—C4—C5	114.1 (4)	C6—N1—C3—C4	5.9 (5)
O1—C3—C7—C8	−149.7 (3)	C6—N1—C3—C7	130.3 (4)
O1—C3—C7—C12	34.0 (5)	C7—C3—C4—C5	−121.0 (4)
C2—C1—N1—C3	−16.7 (4)	C7—C8—C9—C10	0.9 (6)
C2—C1—N1—C6	120.9 (4)	C8—C7—C12—C11	1.0 (6)
C2—O1—C3—N1	−35.7 (4)	C8—C9—C10—C11	−0.4 (7)
C2—O1—C3—C4	−148.3 (3)	C9—C10—C11—C12	0.2 (7)
C2—O1—C3—C7	85.2 (3)	C10—C11—C12—C7	−0.5 (7)
C3—N1—C6—O2	168.1 (4)	C12—C7—C8—C9	−1.1 (5)
C3—N1—C6—C5	−12.2 (5)		

### Hydrogen-bond geometry (Å, º)

*Cg*3 is the centroid of the C7–C12 ring.

**Table:**

*D*—H···*A*	*D*—H	H···*A*	*D*···*A*	*D*—H···*A*
C5—H5*A*···O2^i^	0.97	2.58	3.346 (6)	136
C10—H10···*Cg*3^ii^	0.93	2.88	3.734 (6)	154

Symmetry codes: (i) −*x*+1, *y*−1/2, −*z*; (ii) −*x*+1, *y*−1/2, −*z*+1.
